# Editorial: Leveraging information systems and artificial intelligence for public health advancements

**DOI:** 10.3389/fpubh.2026.1789559

**Published:** 2026-02-06

**Authors:** Chin-Ling Chen, Chenxi Huang, Mueen Uddin, Praveen Neeli, Minglian Qiu

**Affiliations:** 1 Department of Computer Science and Information Engineering, Chaoyang University of Technology, Taichung, Taiwan; 2 School of Informatics, Xiamen University, Xiamen, China; 3 College of Computing and Information Technology, University of Doha for Science and Technology, Doha, Qatar; 4 University of Texas MD Anderson Cancer Center, Houston, TX, United States; 5 The First Affiliated Hospital of Fujian Medical University, Fuzhou, Fujian, China

**Keywords:** artificial intelligence, digital readiness, information systems, public health, public health practices

The integration of Artificial Intelligence (AI) and Information Systems (IS) is fundamentally reshaping the public health landscape, offering groundbreaking solutions for disease prevention, management, and surveillance. As these technologies advance, they offer unprecedented opportunities to improve health outcomes through real-time monitoring, the creation of personalized health strategies, and greater efficiency across health interventions. Despite these promising developments, the public health sector continues to face ongoing questions regarding the optimal implementation of these tools across diverse populations and complex healthcare systems. This Research Topic was established to explore how the fusion of AI and IS can transform and elevate the efficacy of public health practices, policies, and education.

## Assessing health system infrastructure and digital readiness

A primary hurdle in leveraging technology is ensuring that health systems are structurally ready for digital adoption. Two key studies in this Research Topic address this “readiness” from different angles. Snowdon et al. contribute a crucial piece. Their work utilizes a cross-sectional analysis to assess the current capacity of digital public health systems, providing a benchmark for how state-level infrastructures can evolve to meet modern technological demands.

While infrastructure is physical, “literacy” is the human component of readiness. Kumar et al. explore the barriers preventing healthcare professionals and systems from fully embracing AI. Their research emphasizes that the effectiveness of technological tools depends on the literacy of those who deploy them, identifying specific adoption challenges within the public healthcare sector.

## Advancing disease forecasting and chronic disease management

One of the most powerful applications of AI in public health is its ability to predict future crises. Du explores this topic by utilizing information systems to process time-series data. This study demonstrates how proactive forecasting can provide public health officials with the lead time necessary to mitigate outbreaks before they escalate.

In addition to infectious diseases, chronic conditions represent a significant global health burden that requires continuous monitoring. Xie introduces a specialized technical approach. This study demonstrates how sophisticated neural network architectures, such as capsule networks, can improve the accuracy of predictions for long-term health issues in both clinical and community settings. Complementing this specific model is the systematic review by Liu and Wang. Their review synthesizes current research on combining Internet of Things (IoT) devices and machine learning to provide a comprehensive framework for patient monitoring.

## Specialized health interventions: maternal and geriatric care

Tailoring AI and IS to specific life stages is a recurring theme in this Research Topic. Maternal health, for instance, benefits significantly from data-driven management. Li, Zhang et al. present evidence showing that digital logging and risk factor management can directly improve maternal outcomes. Further exploring the psychological aspects of digital health, Zhou et al. conducted a cross-sectional study. Their findings suggest that eHealth literacy does not work in isolation; rather, it boosts a woman's self-efficacy, which in turn enhances her readiness for childbirth.

Fu et al. contribute to this discussion. The proposed Prophet-LSTM model demonstrated superior performance in predicting student mental health risks compared to other machine learning algorithms. Evaluation metrics, including the detection rates for psychological issues and for no psychological issues, confirmed the model's high accuracy.

At the other end of the life span, the aging population requires intelligent systems for better health management. Li, Hou et al. address this issue. By leveraging hypergraph convolution, a complex AI technique, they developed a platform capable of addressing the multifaceted needs of the elderly, helping bridge the gap in geriatric care.

## Behavioral health, physical fitness, and education

Monitoring and influencing health behaviors is another area where AI excels. In educational environments, Lu and Ruijuan explore the use of monitoring systems. This research highlights how AI can recognize physical actions to monitor student health and activity levels within schools.

Cui and Yin address the psychological barriers to physical activity. Their use of explainable AI is particularly noteworthy, as it helps identify why students who intend to exercise often fail to do so, providing actionable insights into behavioral interventions. Similarly, Wang and Liu propose a digital social solution. Their model suggests that sharing health “life logs” among peers can foster a supportive environment that promotes fitness among adolescents. Furthermore, Wu et al. critically analyze the infrastructure. The proposed system demonstrated superior accuracy in recognizing emotional states than existing methods. The attention mechanisms provided interpretability by highlighting the most informative physiological features for emotion classification. Omri et al. investigated the intersection of technology and the labor market. This study raises important questions about equity, examining whether robust governance and higher education can shield vulnerable populations from the potential negative economic impacts of AI.

## Conclusion and future outlook

The 14 articles presented in this Research Topic demonstrate the vast potential of Information Systems and Artificial Intelligence in modernizing public health. From digital maturity assessments in Missouri to secure blockchain transactions for IoT, these studies provide a roadmap for a more data-driven health sector. However, the editor notes that significant challenges remain, particularly regarding data privacy and security, as well as the equitable distribution of these benefits.

The findings in this Research Topic underscore that the successful implementation of AI in public health requires more than advanced algorithms; it also necessitates robust infrastructure, high levels of eHealth literacy, and a commitment to security. By addressing these themes, this Research Topic contributes to a deeper understanding of how these powerful tools can be leveraged to foster a healthier and more resilient society.

